# Effectiveness of oral litholysis therapy for improving glucose intolerance and malnutrition in patients with poor results following endoscopic therapy and extracorporeal shock wave lithotripsy for calcified pancreatic stones

**DOI:** 10.1007/s12328-015-0591-x

**Published:** 2015-08-02

**Authors:** Nobuo Ashizawa, Koichi Hamano, Aiji Noda

**Affiliations:** Division of Gastroenterology, Japan Community Healthcare Organization Tamatsukuri Hospital, Tamayu-cho, Yumachi 1-2, Matsue, 699-0293 Japan; Division of General Medicine, Aichi Medical University Hospital, Karimata, Yazako, Nagakute-cho, Aichi-gun, Aichi 480-1195 Japan; Division of Internal Medicine, Aichi Medical University Medical Clinic, Higashi-ku, Higashisakura 2-12-1, Nagoya, 461-0005 Japan

**Keywords:** Chronic calcific pancreatitis, Pancreatic stones, Impaired glucose tolerance, Oral litholysis therapy, Trimethadione

## Abstract

We report a case of pancreatolithiasis in which glucose intolerance and malnutrition were significantly improved after starting oral litholysis therapy (OLT) with use of trimethadione. A 43-year-old female with multiple calcified stones in the main and peripheral pancreatic ducts had experienced recurrent and severe attacks of pain for 7 years (from 21 to28 years of age). Impaired glucose tolerance was first noted at the age of 32 years. We started OLT after interventional endoscopic therapy combined with extracorporeal shock wave lithotripsy failed because of kink and stenosis of the main pancreatic duct (MPD). Over the next 9 years, a significant decrease in total pancreatic calcified stone volume was shown by computer analysis of follow-up computed tomography images. Larger stones completely disappeared without attacks of pain. In addition, both glucose intolerance and insulin secretion were significantly ameliorated, followed by improvement of malnutrition. OLT may induce intraductal decompression by dissolving stones in the peripheral ducts as well as the MPD, with resulting preservation of endocrine function and improvement of malnutrition. Since the present results were obtained in a single case, further clinical trials are necessary to evaluate the value of performing OLT under various conditions to eliminate stones.

## Introduction

Extracorporeal shock wave lithotripsy (ESWL) as well as endoscopic or surgical therapy are indicated for stones in the larger pancreatic ducts, particularly the main pancreatic duct (MPD), and are reported to be effective for immediate relief of pain. However, many patients show unsatisfactory response to these treatments, leading to recurrence of stones during the follow-up period. In addition, their long-term effects on the recovery of pancreatic dysfunctions remain uncertain [1–4].

Pancreatic diabetes is an important complication in patients with chronic calcific pancreatitis and can reduce quality of life by varying degrees. Overcoming these problems is a primary concern when managing patients with chronic calcific pancreatitis, particularly during the so-called transitional stage. Previously, Noda et al. [5–7] developed oral litholysis therapy (OLT) with use of trimethadione (3,5,5-trimethyl-2,4-oxazolidinedione; TMO) for calcified pancreatic stones and have reported its usefulness for chemical dissolution of pancreatic stones. Here, we report a case of chronic calcific pancreatitis, in which both endocrine dysfunction and malnutrition were gradually but definitely improved during OLT.

## Case report

**Fig. 1:**
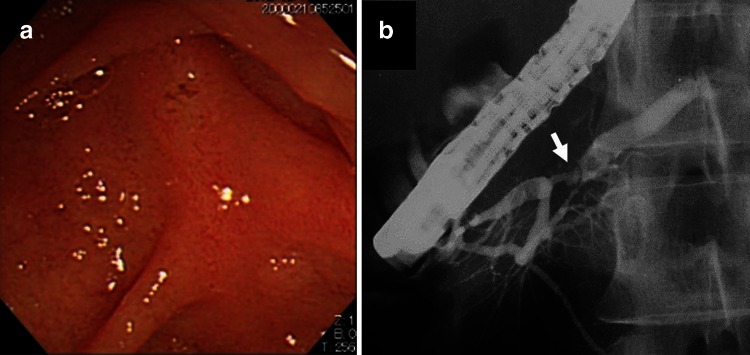
Endoscopic retrograde pancreatography images before OLT. **a** Endoscopic image of the papilla of Vater. There was no abnormal finding. **b** Pancreatographic image. *Arrow* indicates kink and stenosis of the MPD in the head of the pancreas. *OLT* oral litholysis therapy

A 43-year-old female had suffered from recurrent epigastric and back pain for 7 years (from 21 to 28 years of age). At the age of 26, a diagnosis of chronic calcific pancreatitis was made based on findings of hyperamylasemia and multiple pancreatic calcified stones detected by computed tomography (CT). Her past medical history was unremarkable, she was a nonsmoker and did not consume alcohol, and there was no family history of pancreatic diseases or diabetes mellitus. No abnormalities were observed in important organs including the liver, kidneys, and gastrointestinal tract. Blood levels of parathyroid hormone in July 1999 (at 28 years of age) were within normal range [carboxyl-terminal 0.2 (<0.6) ng/ml, intact 24 (14–66) pg/ml, and high-sensitivity 229 (90–270) pg/ml]. There was no endoscopic abnormality of the papilla of Vater (Fig. 1a). Endoscopic retrograde pancreatography revealed kink and stenosis of the MPD in the head of the pancreas (Fig. 1b), which hampered endoscopic insertion of a guidewire to the distal MPD. From October 1999 to November 1999 (at 28 years of age), ESWL was performed six times against the largest stone in the MPD; however, no obvious change was observed in its size and shape. In February 2000 (at 29 years of age), an endoscopic sphincterotomy was insufficiently performed. Thus, the patient did not show a response to interventional endoscopic retrieval therapy combined with ESWL. Although hospitalization had been repeated because of recurrent and severe pain attacks along with temporary hyperamylasemia, the patient has not suffered a pain attack since 1999 (at 28 years of age).

**Fig. 2:**
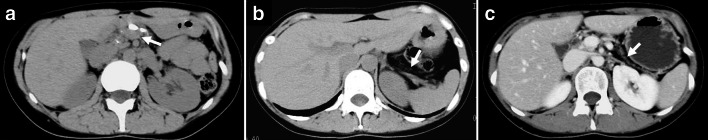
Original images from 3-mm slice abdominal CT scans obtained at 31 (**a**, **b**) and 32 (**c**) years of age (before OLT). **a** Note the multiple calcified stones in the head and body of the pancreas. *Arrow* indicates superior mesenteric vein. **b** *Arrow* indicates markedly atrophic pancreatic parenchyma without calcification in the tail of the gland. **c** Enhanced image showing atrophic parenchyma in the tail of the gland is more distinguishable (*arrow*). *OLT* oral litholysis therapy

CT scanning performed 3 years later (at 31 years of age) revealed multiple pancreatic calcified stones in the head and body, and markedly atrophic pancreatic parenchyma without calcification in the tail (Fig. 2). Magnetic resonance cholangiopancreatography using 1.5 tesla did not visualize the MPD and its side branches in the body and tail. Ultrasonographic images of the pancreatic parenchyma were indistinct because of multiple calcific stones with acoustic shadows. Thereafter, we performed follow-up CT scanning at appropriate intervals to check for parenchymal changes.

**Fig. 3:**
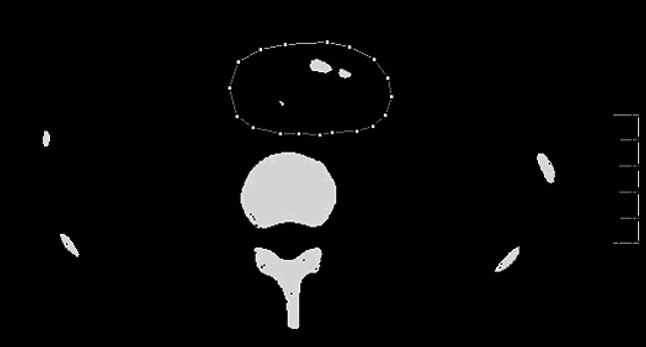
A processed image of original 3-mm slice CT shown in Fig. 2a using the image processing and analysis component ‘Sync Measure 3D’ in the Image J software package. In the *polygonal lined area* indicating pancreatic territory, *white areas* indicate lesions shown by voxels numbering >33,006 (CT radiodense unit used in ‘Sync Measure 3D’). The total size of the *white areas* in the pancreas (33,006 threshold-positive lesions) was calculated to measure the pancreatic calcified stones in each 3-mm slice CT image. *OLT* oral litholysis therapy

Using computer analysis of obtained CT images, we attempted to estimate the pancreatic calcified stone volume. The CT device used during the first period of this study (July 2002 to December 2005) was a single-slice CT scanner (X-Vigor Single-Slice CT; Toshiba Medical Systems Corporation, Japan), while that used during the second period (January 2007 to December 2013) was a multi-slice CT scanner (Asteion Super 4 Edition Multi-Slice CT 4 Data Acquisition System; Toshiba Medical Systems Corporation). The single-slice CT images were not <3 mm in size. Thus, in order to obtain consistent follow-up findings in examinations to determine pancreatic calcified stone volume, we used 3-mm slice CT scan images produced from voxel data of the same size obtained by both devices. We measured the total volume of voxels with a greater number than the determined threshold in CT images of pancreatic territories (threshold-positive voxel lesions) obtained using the image processing and analysis software package ‘Sync Measure 3D’, which is part of the Image J software package (Fig. 3). The number of voxels in a CT image is a unique radiodense unit in the ‘Sync Measure 3D’ package. Data showing each threshold-positive voxel volume suggested that the detectors of the single-slice CT device had a lower sensitivity for a high number of voxels and produced increased noise in images with a low number of voxels compared to the multi-slice CT device. For single-slice CT scanning, the threshold with the highest signal/noise ratio was 33,006, which was selected as appropriate for obtaining follow-up data regarding pancreatic calcified stone volume. Furthermore, for follow-up data regarding pancreatic parenchymal volume, we traced the outline of the head of the pancreas on the original CT scan image and measured its volume using the ‘Sync Measure 3D’ program. Tracing of the body and tail of the gland was difficult because of the markedly atrophic change of the parenchyma (Fig. 2b). The head of the pancreas was defined as the area to the right of the left border of the superior mesenteric vein.

**Fig. 4:**
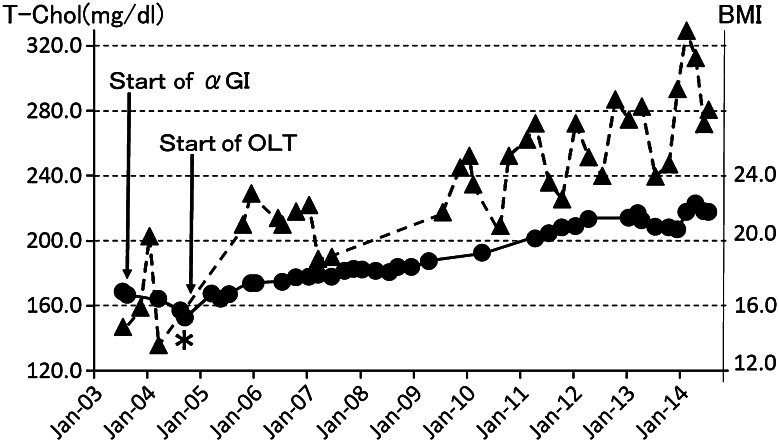
Follow-up data for serum total cholesterol (T-Chol, *filled triangle*) and body mass index (BMI, *filled circle*). Minimum BMI was 15.2 in September 2004 (*asterisk*), and then gradually increased after starting OLT. Serum total cholesterol also showed a tendency to increase and hypercholesterolemia recently appeared. *αGI* α-glucosidase inhibitor. *OLT* oral litholysis therapy

**Fig. 5:**
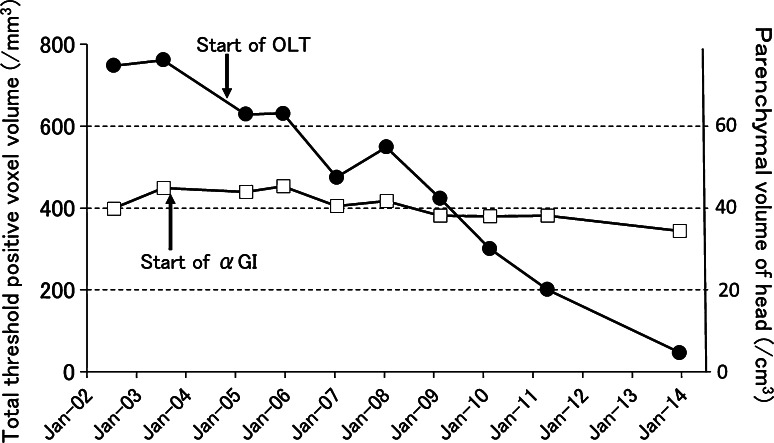
Follow-up data for 33,006 threshold-positive voxel volume in the pancreas (*filled circle*) and parenchymal volume of the head of the pancreas (*open square*). *αGI* α-glucosidase inhibitor. *OLT* oral litholysis therapy

**Fig. 6:**
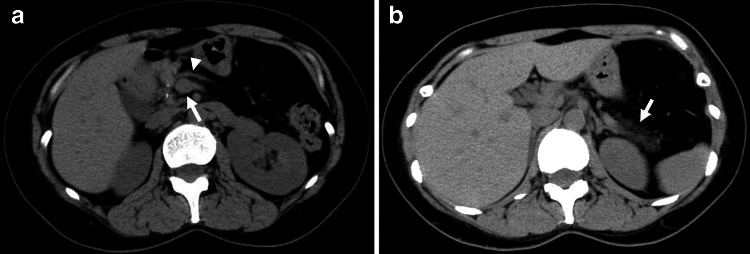
Images from 3-mm slice abdominal CT scans obtained in December 2013, 9 years after starting OLT (at 42 years of age). **a** *Arrowhead* indicates the markedly atrophic parenchyma in the body of the pancreas. *Arrow* indicates the superior mesenteric vein. Note the complete disappearance of larger stones in the head and body of the gland vizualized before OLT (Fig. 2a). **b** *Arrow* indicates markedly atrophic parenchyma without calcification in the tail of the gland, showing almost the same severity as observed before OLT (Fig. 2b). *OLT* oral litholysis therapy

During the follow-up period, there were no pain attacks and postprandial plasma glucose (PG) reached the highest level of 196 mg/dl in July, 2003 (at 32 years of age). The level of hemoglobin A1c increased from 5.2 % in August 2000 (at 29 years of age) after ESWL to 5.9 %. Furthermore, the initial 75-g oral glucose tolerance test (75-g OGTT) performed at almost the same time showed impaired glucose tolerance with insulin resistance accompanied by delayed response of immunoreactive insulin (IRI) and a decreased insulinogenic index (ΔIRI/ΔPG) of 0.37 at 30 min (Table 1). In order to prevent progression to overt diabetes, we started administration of an α-glucosidase inhibitor (αGI) together with double the usual dose of pancreatic enzyme. However, the second 75-g OGTT performed in May 3004, 10 months after the start of αGI (at 33 years of age), showed further deterioration of glucose tolerance and a decrease in insulin secretion (insulinogenic index 0.29) (Table 1). The patient’s body weight and body mass index (BMI) were lowered to nadirs of 34.8 kg and 15.2, respectively, in September 2004 (at 33 years of age) (Fig. 4, asterisk). As the patient preferred aggressive but less invasive treatment to prevent deterioration of glucose tolerance and malnutrition, we selected OLT with approval of the Ethics Committee of Aichi Medical University. Before starting treatment, a signed informed consent form was obtained from the patient in accordance with the Helsinki Declaration of the World Medical Association. Before starting OLT in October 2004 (at 33 years of age), no macroscopic steatorrhea was observed, and there were no abnormalities in blood cell count, blood chemistry (except the glucose metabolism), and tumor markers including elastase 1, CA19-9, Dupan-2 and Span 1. TMO was given orally at a starting dose of 0.6 g/day for 3 months, followed by 0.9 g/day, which is the recommended dose for adults [5]. After starting OLT, follow-up findings for the threshold-positive voxel volume demonstrated a gradual and marked decrease, with the exception of two mild temporal increases, while the parenchymal volume of the head of the gland showed a slight decrease (Fig. 5). At 9 years after starting OLT, CT images obtained in December 2013 demonstrated complete disappearance of the larger stones in the head and body of the pancreas (Fig. 6a) and markedly atrophic parenchyma in the body and tail of the gland, showing almost the same severity as observed before the treatment (Fig. 6b).

**Table 1:** Follow-up data of 75-g oral glucose tolerance test

Date	PG (mg/dl)					IRI (IU/ml)					Insulinogenic index
	0 min	30 min	60 min	90 min	120 min	0 min	30 min	60 min	90 min	120 min	(ΔIRI/ΔPG at 30 min)
July 2003	99	176	177		171	5.1	33.3	51.9		92.4	0.37
August 2003	Start of αGI										
May 2004	92	157	195		198	4.0	23.0	32.8		69.9	0.29
October 2004	Start of OLT										
May 2005	93	157	165	156	133	5.0	48.3	61.7	70.6	69.0	0.55
April 2006	98	158	172	150	157	5.1	51.3	81.7	91.0	103.8	0.77
May 2007	92	155	205	173	131	4.0	58.6	90.1	115.0	118.0	0.87
April 2008	92	155	181	149	130	5.2	40.1	54.3	48.7	34.7	0.55
April 2009	95	179	172	122	101	2.8	28.5	42.7	50.6	45.2	0.31
April 2010	90	149	143	160	127	2.5	43.6	39.1	47.1	43.6	0.70
April 2011	93	138	128	141	149	7.1	48.6	29.6	32.2	44.9	0.91
April 2013	99	174	198	199	133	6.5	42.0	65.2	93.0	72.3	0.47
July 2014	102	182	211	221	172	7.1	48.4	53.9	116.0	128.0	0.52

*PG* plasma glucose, *IRI* immunoreactive insulin, *αGI* α-glucosidase inhibitor, *OLT* oral litholysis therapy

After starting OLT, the PG level gradually decreased, whereas the IRI level gradually increased during the first 2.5 years (until May 2007) and then decreased with improvement of the initial secretory response (Table 1). In April 2011 (6.5 years after starting OLT), peak IRI was 48.6 μU/ml, the same level as observed in the non-obese healthy Japanese population, and the insulinogenic index was improved to 0.91 (Table 1). In addition, the patient’s appetite gradually increased, which induced an increase in BMI to 22.8 (April 2014) and the appearance of hypercholesterolemia (Fig. 4). A recent 75-g OGTT (performed in July 2014) showed an increase in PG at 60 and 90 min in spite of the increase in IRI (delayed response) (Table 1), and hemoglobin A1c was 6.0 % in September 2014. The result of the BT-PABA (N-benzoyl-L-tyrosyl-*p*-aminobenzoic acid) test was 85.5 %/6 h (<70.0 %/6 h) before OLT (in September 2004) and showed a normal or almost normal level during OLT, ranging from 68.7 %/6r to 83.4 %/6r.

No laboratory findings suggesting TMO toxicity were noted in the liver, kidneys, blood, skin, etc. The patient did not complain of photophobia, which is known to be the primary adverse effect of TMO. No abnormality was found in her eyes. At present, the therapy is being continued to dissolve residual stones, as well as prevent their enlargement or the recurrence of stones.

## Discussion

Pancreatic stones can act as an intraductal barrier to elevate intraductal pressure, evoke pain [8], and deteriorate pancreatic functions. In experimental models of chronic obstructive pancreatitis, increased pancreatic duct pressure was found to evoke various kinds of organic and functional changes of the pancreas, including tissue edema, increase in fibrous tissue, deletion of acinar cells, reduction in pancreatic microvascular blood flow (PMBF) [9, 10], and glucose intolerance in spite of preservation of the islets of Langerhans [11]. Pancreatic duct decompression in such experimental models was also found to induce regeneration of acinar cells and improvements in pancreatic edema, fibrosis, and PMBF [9, 10], which restore the function of preserved endocrine cells.

ESWL, as well as endoscopic and surgical therapy, has been reported to be effective for removal of stones in larger pancreatic ducts and immediate relief of pain [1–4]. However, these treatments frequently result in poor outcome for patients with diffuse or multiple stones. A multicenter study conducted in Japan revealed that the rate of recurrence after combined ESWL and endoscopic therapy was 22.0 %, and was especially increased in patients with MPD stenosis [4]. Their long-term effects on recovery of pancreatic dysfunctions remain uncertain [1–4]. We think these therapies have little efficacy against the increasing number of small stones in peripheral pancreatic ducts, which cause silent and gradual deterioration of endocrine and exocrine functions despite the removal of MPD stones. Other means should be introduced to preserve pancreatic parenchyma and functions as much as possible by preventing precipitation and growth of calcium carbonate and dissolving immature or small stones, especially in peripheral pancreatic ducts. These effects, if obtained, may also save patients from recurrent attacks.

This case can be considered as idiopathic chronic pancreatitis. Stenosis of the MPD may play an etiological role in the development of chronic inflammation and calcification of concretions. Before starting OLT, this patient had been in a state of glucose intolerance and malnutrition but without evidence of definite pancreatic exocrine dysfunction as shown by steatorrhea and abnormal BT-PABA test. This indicates that chronic pancreatitis in this patient had been in the transitional stage and not in the noncompensatory stage. Both kink and severe stenosis of the MPD, which had formed in the head of the pancreas (Fig. 1b), hampered endoscopic therapy and ESWL. As the findings of the second OGTT strongly suggested a high risk of progression to overt diabetes, we applied OLT with use of TMO as an attempt to preserve pancreatic function or delay its aggravation. TMO, an older antiepileptic agent that is rarely used in epileptic patients because more effective drugs are available, is converted to dimethadione (5,5-dimethyl-2,4-oxazolidinedione; DMO) by metabolic demethylation. DMO is a lipid-soluble weak organic acid with a molecular weight of 129.12 and pKa of 6.13 at 37 °C that diffuses rapidly to alkaline fluid compartments in accordance with pH gradient [12]. Macroautoradiology using [^14^C]DMO in the rat revealed significant accumulation of DMO in the pancreas [13]. Furthermore, the non-dissociated form of DMO diffuses from capillaries to alkaline pancreatic juice via extracellular space [5] and dissolves calcium carbonate, a main constituent of pancreatic stones, by releasing proton in pancreatic juice [14].

The prior interventional therapies including endoscopic sphincterotomy and papillary dilatation combined with ESWL may facilitate the effect of OLT on stone elimination [7]. In this patient, endoscopic sphincterotomy was insufficient, and the papilla of Vater was endoscopically normal (Fig. 1a). To date, we have never experienced spontaneous dissolution of pancreatic stones, and there is no evidence to indicate the direct effect of DMO on endocrine cells related to glucose metabolism. Therefore, we stress that OLT is responsible for the results obtained in this patient. The following mechanism can be induced on referring to the results obtained in experimental models of chronic obstructive pancreatitis [9–11]. OLT can dissolve immature or small calcified stones in peripheral ducts as well as larger ducts, and decompress intraductal pressure. These effects can improve organic changes including pancreatic edema, fibrosis and PMBF, and ameliorate the malnutrition and dysfunction of preserved endocrine cells. The most recent result of OGTT in this patient showed hypersecretion of insulin and delayed response, which is a pattern of insulin resistance largely attributable to excessive recovery from malnutrition (i.e., the so-called tendency of metabolic syndrome).

Thus, oral administration of TMO shows a steady effect on calcified stones in the peripheral pancreatic ducts as well as the MPD, although long-term use of the recommended dose of TMO is needed to dissolve stones and prevent relapse of stones [5, 6]. In the present case, two mild temporal increases of threshold-positive volumes were observed in 3-mm slice CT scan images in spite of a steady decrease in pancreatic calcified stone volume, which may have been the result of aggregation or separation of stones. It is considered that threshold-positive volumes from 1-mm slice CT scan images can provide more precise information regarding changes in total and peripheral pancreatic duct stone volume.

It is generally known that 75-g OTT is extremely useful for evaluation and follow-up of glucose tolerance. In this case, test results provided much useful data regarding changes in PG and insulin secretion. We recommend that both 75-g OGTT and computer analysis as mentioned above be applied regardless of the removal therapy utilized for pancreatic stones.

OLT may elicit a decrease in intraductal pressure by dissolving stones in peripheral ducts as well as the MPD, which may have a favorable effect on endocrine function and contribute to improvement of malnutrition. Since the present results were obtained in a single case, further clinical trials are necessary to evaluate the value of performing OLT under various conditions to eliminate stones.
